# Metal Nanoparticles: Thermal Decomposition, Biomedicinal Applications to Cancer Treatment, and Future Perspectives

**DOI:** 10.1155/2018/9354708

**Published:** 2018-04-18

**Authors:** Ayodele Temidayo Odularu

**Affiliations:** Department of Chemistry, University of Fort Hare, Private Bag X1314, Alice 5700, South Africa

## Abstract

Monodispersed forms of metal nanoparticles are significant to overcome frightening threat of cancer. This review examined pragmatically thermal decomposition as one of the best ways to synthesize monodispersed metal nanoparticles which are stable and of small particle sizes. Controlled morphology for delivery of anticancer agent to specific cells can also be obtained with thermal decomposition. In addition to thermal decomposition, the study also looked into processes of characterization techniques, biological evaluation, toxicity of nanoparticles, and future perspectives.

## 1. Introduction

Cancer is a worldwide disease [1–3]. A report from the National Cancer Institute (NCI), titled “Statistics at a Glance: The Burden of Cancer Worldwide,” said, “cancer was among the global leading fatality with 14.1 million cases which emerged in 2012 alongside with cancer-related issues of 8.2 million globally” [4]. It is the second cause of fatality after heart disease with 8.8 million lives affected in 2015, of which 70% came from low- and middle-income countries [5, 6]. World Health Organization (WHO) predicted the number of new cases of cancer disease to rise to 70% over the next twenty years [6]. In addition to this, alarming rates at which cancer subjects experienced resistance to antineoplastic drugs and challenges of its side effects when these drugs were administered, call for an alternative drug. A current approach to deal with the resistance and side effects can be drawn from nanotechnology. Nanotechnology is made up of small materials of magnitude between 1 and 100 nm, containing structures with arrangements of atoms, popularly referred to as nanoparticles [7]. It includes disciplines, such as chemistry, computing, electronics, energy, engineering, physics, and *biomedicine* [8]. In biomedicine, nanomedicine (medical application of nanotechnology) has a promising approach to detect and treat cancer [9, 10]. The focus of nanomedicine is to pave an effective way in the health sector to rid it of dangerous diseases, such as cancer [11]. Zainal et al. reported that one eminent way nanomedicine helped in going about this was through inorganic nanoparticles [12]. Nanoparticles enhance the delivery of promising anticancer agents on malignant cells [13]. It is also used as a anticancer therapeutic agent, as well as, to detect and diagnose cancer with magnetic resonance image (MRI) [9]. The goal of a better delivery system for drugs has made intense research to be delved in nanoparticles over the past ten years [12]. The greatest challenge in the research of nanotechnology is how to obtain a controlled nanometric size and shape. Nanoparticles with their small sizes when compared to bulk materials have helped to procure hopes with their edge advantages of *quantum size effect* and *high surface area to volume ratio* [13–15]. Uniqueness of inorganic nanoparticles due to the small size and morphology reflects optoelectronic characteristics [11, 12]. Monodispersed metal nanoparticles by thermal decomposition can be used to overcome resistance and side effects of the conventional drugs for cancer. The impact of monodispersed metal nanoparticles on cancer was considered in this study. This scientific communication also addressed synthesis, reaction method of coordination compounds, characterization, prevention of polydispersed, and toxicity of nanoparticles. The aim of this review was to look at the application of thermal decomposition as the most appropriate method of synthesis to obtain monodispersed forms of metal nanoparticles.

### 1.1. Reaction Methods prior to Synthesis of Metal Nanoparticles

Prepared coordination compounds are right precursors to synthesis of nanoparticles because they had been confirmed to be an effective path to give high-quality monodispersed metal nanoparticles [16]. Kelly et al. reported that coordination compounds and metallocenes were convenient precursors [17]. Further treatment of these coordination compounds before thermal decomposition involves the use of stabilizers and capping agents.

#### 1.1.1. Reducing Agents (Stabilizers/Scavenging Agents)

Reducing agents are inorganic and organic compounds used to prepare metal nanoparticles by decreasing oxidation states of metallic ions in coordination compounds or metallic salts to zero. They are also used to prevent agglomeration of metal nanoparticles [13, 18, 19]. Reducing agents are referred to as stabilizers or scavenging agents. Effective stabilizers can be natural polymers, (chitosan and oligochitosan) or artificial polymers (alginate, poly vinyl alcohol (PVA), and polyvinyl pyrrolidone (PVP)). Both forms of polymers have functional groups, such as amino (-NH_2_), carboxylic acid (-COOH), hydroxyl (-OH), and thiols (SH) [18]. Natural polymers support green chemistry. They are sometimes used as capping agents for nanoparticles. The process of providing electrons or radicals to metallic ions allows them to be referred to as scavenging agents. They can be classified as weak and strong stabilizers. Weak reducing agents support slow reaction, thereby allowing particles to grow over a long period of time to give a faceted and less than one nanometer nanoparticles. Examples are sodium citrate and potassium bitartraate [19]. On the other hand, strong reducing agents, such as formamide and ortho-anisidine form bigger and spherical nanoparticles [19]. Other reducing agents which are very useful and not polymers are long chain organic molecules, sodium borohydride, and ethylene glycol.

#### 1.1.2. Surfactants (Capping Agents) and Colloidal Nanoparticles

Surfactants are protective and surface acting agents which further prevent agglomeration by avoiding interaction of nanoparticles with one another [13]. They stabilize the nanoparticles formed by reducing agents, thereby allowing some researchers to refer to them as stabilizing agents. The qualities of soft-temperate model, capability to transform chemical kinetics, and easy maneuverability possessed by surfactants allow them to control morphologies of nanomaterials [20]. They are also referred to capping agents. During thermal decomposition, the reduced samples are injected inside the surfactants at a certain temperature, the colour of the sample changed to black after a while in an inert environment. Black solution is an indication of colloidal nanoparticles [21]. As it applies to stabilizers, polymers and functional groups of amines, carboxylic acids, hydroxyls, and thiols are good capping agents for successful thermal decomposition [13].

#### 1.1.3. Dual Stabilizing and Capping Agents

In some cases, researchers use neither stabilizing nor capping agents. In other cases, some compounds, such as sodium citrate can act as both a stabilizing and a capping agent [13].

### 1.2. Synthesis of Metal Nanoparticles

Two main approaches of synthesizing metal nanoparticles are top down and bottom up [22]. Both approaches have their advantages and disadvantages. Imperfection observed on the surfaces and damage of crystals of nanostructures is the biggest challenge in the top-down approach of synthesizing nanoparticles [22, 23]. This approach is still in use despite this challenge. Method used for synthesis of nanoparticles has a great impact on small particle size and morphology. Three different methods of synthesizing nanoparticles are physical (microwave irradiation, sonochemical, ultraviolet radiation, laser ablation, thermal decomposition (thermolytic), photochemical, or radical induced), chemical (supercritical fluid, coprecipitation, use of inorganic matrix as support, and organic solvents), and biological (use of algae, bacteria, fungi, or plants) [10–13, 24]. From the three methods highlighted, this review focused on the physical method of thermal decomposition because other methods are polluting, time consuming, forming agglomerated and aggregated nanoparticles with wide distributed sizes, and very expensive [15, 22]. Thermal decomposition is an innovative method to synthesize stable monodispersed nanoparticles product [21]. It is a research area that is fast developing, clearer, and economical when compared with conventional methods [25]. It is also one of the easiest and the most convenient way to synthesize monodispersed metal nanoparticles [26]. In addition, it answers the greatest challenge of obtaining a controlled nanometric size and shape in nanotechnology research [27]. Other factors to be considered to obtain controlled nanometric size are duration of synthesis, temperature, concentrations of the reactants, stabilizers, capping agents (surfactants), and types of surfactants [28]. Palacious-Hernández et al. and Kino et al. explained that the solventless method of thermal decomposition was an easy and moderate route which required no raw material [27, 28]. On a similar note, Tran et al. stated that thermal decomposition allowed large amount of nanoparticles to be produced once, unlike biological method which produced small amount of nanoparticles [26]. Tran et al. also emphasized the relevance of the precursor injection method, where the precursor was injected into hot solution of the surfactant [26]. This was to induce rapid nucleation of nanoparticles with small sizes which are either the same or similar and possess narrow size distributions [26]. These monodispersed nanoparticles could also be referred to as being homogeneous [22].

#### 1.2.1. Thermal Decomposition

Factors such as nature of metallic ion and the force of reaction with the ligands in coordination compounds have effects on the temperature and pressure at which thermal decomposition takes place [17]. In other words, coordination compounds can be thermodynamically stable or kinetically stable. No particular stabilizing agent (stabilizer) is used for thermal decomposition. With regard to stabilizers, Rao et al. reported that capping agents, such as carboxylic acids and alkyl amines, influenced formation of monodispersed nanoparticles obtained from thermal decomposition [29]. The overall effect has an impact on achieving monodispersed nanoparticles [17].

### 1.3. Washing and Drying of Nanoparticles

After thermal decomposition, the reaction vessel is cooled at room temperature in a switched-on fume cupboard so as to lower the temperature [21, 28]. The switch is ensured to be put off after cooling. Nanoparticles can easily form precipitates in cold polar solvents, such as deionized water, ethanol, and methanol [21]. These solvents help to remove excess reaction materials (capping agents) [21]. The process of centrifugation as the next step separates the precipitates from colloidal nanoparticles. It also cleans the nanoparticles, but excess cleaning causes agglomeration of the nanoparticles. This process is completed when there is homogeneity in the precipitates. Several times of redispersion in nonpolar solvents like benzene, hexane, and toluene purify the precipitates and this signifies a nanoparticle surfactant core-shell structure [21, 28]. Drying of nanoparticles takes place by alcohol drying (chemical extraction), freeze drying (nonthermal), or in vacuum oven (thermal) for a temperature of less than 100°C and for a specific period of few hours or overnight after washing [18, 30–32].

### 1.4. Behaviour and Characterization of Nanoparticles

Most researchers studied the behaviour of nanoparticles using relevant techniques for appropriate characterizations. Wostek-Wojciechowska et al., Dallas et al., and Jung et al. stated that thermal decomposition could be performed as either thermolysis or pyrolysis [33–35]. Dallas et al. did the pyrolysis of his silver nanoparticles at a temperature of 300°C [34]. They also carried out thermolysis in both solid and liquid states [34]. In the case of Wostek-Wojciechowska et al., they reported that better nanoparticles were obtained when in solution than in the solid state [33]. In order to measure the level of thermal stabilities of thermolysed nanoparticles, thermogravimetric analysis is needed. Dallas et al and Khalil et al observed the temperature of nanoparticles with thermogravimetric analysis (TGA) and differential thermal analysis (DTA) [34, 36]. All the aforementioned authors reported temperatures below 300°C for the thermogravimetric analysis. They were all in line with Iravani et al., who reported that a temperature of an less than or equal to 300°C provides a broad range of reaction temperature and permits the effective control of nanoparticles by the variance in the heating temperature while the solvent is left constant [13].

Spectroscopic characterization techniques involve Fourier-Transform Infrared (FT-IR) Spectroscopy (FT-IR), Ultraviolet-Visible (UV-Vis) Spectroscopy, Florescence Spectroscopy (photoluminescence), and Raman Spectroscopy. The FT-IR functions to identify functional groups in the bulk materials and nanoparticles. For the UV-Vis spectroscopy, it supports results from other spectroscopic characterization techniques, thereby giving the geometry and optical properties of the sample.

The optical property of metal nanoparticles requires the band gap [37]:

(1) $$\begin{array}{c} E_{g}\left(\text{Ev}\right)=\frac{1240}{k}\left(\text{nm}\right), \end{array}$$

where *E*_g_ is the band gap energy and *k* is the absorption edge.

Florescence spectroscopy often referred to as photoluminescence spectroscopy because the same instrument does the analyses of both techniques. Both provide the optical properties of the sample. Raman spectroscopy does the identification of the crystal structure and supplementary confirmation of phase purity of prepared samples of nanoparticles [38]. 

In addition to the aforementioned techniques, Mass Spectrometry (MS) and X-ray Photoelectron Spectroscopy (XPS) are important characterization techniques in Materials Chemistry. The MS measures the characteristics of charged particles of individual molecules by converting them into ions, while XPS analyzes surface and interface conditions of materials.

Other important techniques in Materials Chemistry are microscopic analyses, purity check, x-ray diffraction, magnetic characterization, and surface area. Microscopic analyses include Scanning Electron Microscopy (SEM), Transmittance Electron Microscopy (TEM), and Atomic Force Microscopy (AFM). The metal nanoparticles are always characterized for their morphologies using techniques of SEM and TEM. The SEM produces morphology of a sample based on scattered electrons to give images of three dimensional, such as cylinders. In the case of TEM, it produces morphology of a sample based on transmitted electrons to give images of two dimensional, such as thin sheets and thin wires. Atomic Force Microscope (AFM) observes the inside of materials directly.

Elemental Analysis (EA) and Electron Dispersive Spectroscopy (EDS) are used to check for the purity of bulk material and metal nanoparticles respectively.

X-ray diffraction can either be single crystal X-ray diffractometry or powder X-ray diffractometry (XRD). Single crystal X-ray diffractometer analyzes the complete structure of crystalline materials, from the range of simple inorganic solids to complex macromolecules.

The XRD is used to obtain data for the crystalline shape, crystallite size, and orientation in polycrystalline of powdered solid samples from the Scherrer equation:

(2) $$\begin{array}{c} D=\frac{0.9\lambda }{\beta \cos\theta_{\beta }}\theta , \end{array}$$

where *D* is the crystalline size of the metal nanoparticles; *λ* is the wavelength of X-ray radiation; *β* is the full width at half maximum (FWHM) of the diffraction peak; and *θ*_*β*_, the Bragg diffraction angle [37].

The magnetic characterization includes Nuclear Magnetic Resonance (NMR), Electromagnetic Resonance (ESR) and Magnetic Susceptibility Sensors (MSS). The NMR as a scientific technique is employed in the study molecular physics, crystals, and non-crystalline materials, mostly for paired electrons. Nuclear magnetic resonance is used as diagnostic tool to confirm synthesized nanoparticles have specific moieties. The ESR, often called Electromagnetic Paramagnetic Resonance (EPR), provides information on the geometry of the radical and the orbital of the unpaired electron, while MSS provides quantitative measure to which a material may be magnetized in a magnetic field.

The surface area of nanoparticles can be determined using a mathematical expression called Brunauer-Emmett-Teller (BET) as shown in equations (3) and (4). The colloidal state of stable monodispersed metal nanoparticles can be determined using electrophoretic light scattering technique for its zeta potential [39]. Phase of *in vitro* testing prior to *in vivo* testing is a preliminary stage used to test the anticancer activities of the nanoparticles.

(3) $$\begin{array}{c} S_{\text{total}}=\left(\frac{v_{m}Ns}{V}\right), \end{array}$$

(4) $$\begin{array}{c} S_{\text{BET}}=\left(\frac{S_{\text{total}}}{a}\right), \end{array}$$

where *v*_*m*_ is the unit of the monolayer volume of adsorbate gas, *N* is the Avogadro number, *s* is the adsorption cross section of the adsorbing species, *V* is the molar volume of the absorbing species, and *a* is the mass of the adsorbent.

### 1.5. Prevention of Polydispersed Nanoparticles

**Figure 1:**
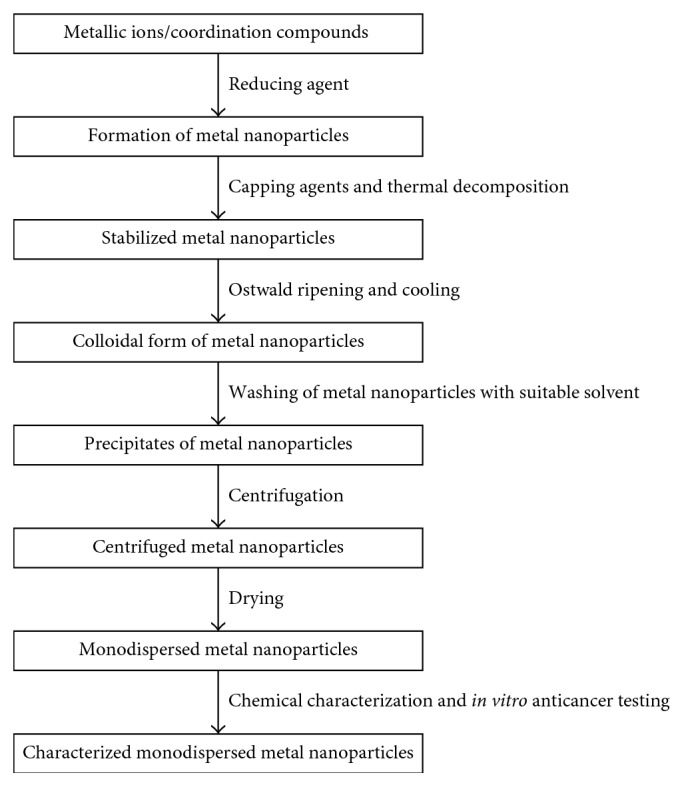
Schematic diagram for the synthesis of monodispersed metal nanoparticles.

Four important steps in chemical synthesis of nanoparticles which aid particle size and uniformity are nucleation, growth of the colloidal particles, Ostwald ripening, and stabilization. During the stage of particle growth, it is possible to control the uniformity of the particle size. This is because once the reaction proceeds to Ostwald ripening, polydispersed nanoparticles are formed rather than monodispersed nanoparticles. Ostwald ripening is a slow diffusion-organized process, also called *second phase coarsening* [40]. It is defined as the dissolving of small nanoparticles and their redeposition on larger particles [41]. Apart from the dispersion of nanoparticles in liquid phase which promotes the formation of polydispersed nanoparticles, processes of drying in powdery form rather than in colloidal slurries form and sintering are also factors which contribute to polydispersity [30]. The process of sintering involves the heating of nanoparticles in powdery form (nanopowders) in order to make them solid [30]. It is high-temperature dependent [30] and, therefore, contrary to the temperature required for monodispersity. Schematic diagram for the synthesis, characterization, and *in vitro* anticancer testing of monodispersed metal nanoparticles is shown in Figure 1.

### 1.6. Toxicity of Nanoparticles

Small sizes of nanoparticles indicate that they are readily absorbed in human body than large sizes [42–44]. The absorption can be through ingestion, inhalation, injection, and transdermal delivery [44–47]. They are more toxic to the health of human beings than large sizes [48]. Research into toxicology of nanoparticles is still in its infant stage [44, 47]. The use of carbon nanotubes depending on the size, uses of silica, biodegradability or polymeric nanoparticles can reduce the toxicity of nanoparticles [48]. There are a number of approaches used to assess the toxicity of nanoparticles [48]. The most cost-effective way which manages time approach is *in vitro* studies [48]. The generally assessed study is cell viability with examples such as biomarkers for apoptosis, cell membrane integrity with lactase dehydrogenase (LDH) assay, comet assay for genotoxicity, immunohistochemistry, and tetrazolium reduction assays [48]. Viable cells are detected using colorimetric assays such as 3-(4,5-dimethylthiazol-2-yl)-2,5-diphenyltetrazolium bromide (MTT), 2,3-bis-(2-methoxy-4-nitro-5-sulfophenyl)-2H-tetrazolium-5-carboxanilide (XTT), 3-(4,5-dimethylthiazol-2-yl)-5-(3-carboxymethoxyphenyl)-2-(4-sulfophenyl)-2H-tetrazolium, and water-soluble tetrazolium salts (WSTs) [48].

## 2. Conclusion

This mini review demonstrated thermal decomposition as the most suitable to synthesize stable monodispersed nanoparticles to order to achieve the goal of cancer treatment in the health sector. Thermal decomposition is easy and economical. Instrumental method of thermogravimetric analysis supported the measurement of the rate of decomposition while the stability as applied to biological application is done using zeta potential. Other relevant characterization techniques help in the identification. Assessments of the toxicity levels are done using *in vitro* assays.

## 3. Future Perspectives

Challenges of agglomeration of metal nanoparticles warrant ways to support thermal decomposition. Method of etching synthesis of metal nanoparticles will be encouraged to support thermal decomposition for the ease of preparing monodispersed nanoparticles. Melting points of samples of synthesized nanoparticles will be used to ascertain the temperature at which thermal decomposition will take place. Solubility test of nanoparticles will be done to detect toxicity and biomembral penetration. Green chemistry promotes good synthesis of nanoparticles in the areas of solvents, reducing agents, and capping agents; therefore, green compounds will be considered. Reference materials will also be considered to assess the efficiency of instruments and methodology relevant to characterization of nanoparticles generally used in preclinical biomedical research such as *in vitro* and interlaboratory proficiency testings. Results from zeta potential for colloidal stabilities of nanoparticles will introduce them to further testing of *in vivo* anticancer testing.

## References

[1] Lotufo P. A. (2015). Smoking and cancer: Brazil and the global burden of disease initiative. *Sao Paulo Medical Journal*.

[2] Jemal A., Bray F., Center M. M., Farlay J., Ward E., Forman D. (2011). Global cancer statistics. *CA: A Cancer Journal for Clinicians*.

[3] Fitzmaurice F., Dicker D., Pain A. (2015). The global burden of cancer 2013. *JAMA Oncology*.

[4] Torre L. A., Bray F., Siegel R. L., Ferlay J., Lortet-Tieulent J., Jemal A. (2015). Global cancer statistics 2012. *CA: A Cancer Journal for Clinicians*.

[5] Ílem-Özdemir D., Gündoğdu E., Elinci M., Aşikoğlu M. (2016). Nanoparticles: from diagnosis to therapy. *International Journal of Medical Nano Research*.

[6] World Health Organization (2017). *Cancer Fact Sheet*.

[7] Bhattacharya R., Mukherjee P. (2008). Biological properties of “naked” metal nanoparticles. *Advanced Drug Delivery Reviews*.

[8] Singh S., Singh A. (2013). Current status of nanomedicine and nanosurgery. *Anesthesia: Essays and Researches*.

[9] Cherukuri P., Glazer E. S., Curley S. A. (2010). Targeted hyperthermia using metal nanoparticles. *Advanced Drug Delivery Reviews*.

[10] Chaturvedi S., Dave P. N., Shah N. K. (2012). Applications of nano-catalyst in new era. *Journal of Saudi Chemical Society*.

[11] Bhattacharyya S., Kudgus R. A., Bhattacharya R., Mukherjee P. (2011). Inorganic nanoparticles in cancer therapy. *Pharmaceutical Research*.

[12] Zainal N. A., AbdShukor S. R. A., Azwana H., Rasak K. A. (2013). Study on the effect of synthesis parameters of silica nanoparticles entrapped with rifampicin. *Chemical Engineering Transactions*.

[13] Iravani S., Korbekandi H., Mirmohammadi S. V., Zolfaghari B. (2014). Synthesis of silver nanoparticles: chemical, physical and biological methods. *Research in Pharmaceutical Sciences*.

[14] Logeswari P., Silambarasan S., Abraham J. (2013). Ecofriendly synthesis of silver nanoparticles from commercially available plant powders and their antibacterial properties. *Scientia Iranica*.

[15] Khalaji A. A. D., Malekan F. (2015). Synthesis and characterization of nanowires hausmannite (Mn_3_O_4_) by solid-state thermal decomposition. *International Journal of Nano Dimension*.

[16] Moloto N., Revaprasadu N., Moloto M. J., Brien P. O., Raftery J. N. (2009). N’-diisopropylthiourea and N,N’-dicyclohexylthiourea zinc(II) complexes as precursors for the synthesis of ZnS nanoparticles. *South African Journal of Science*.

[17] Kelly C. H. M., Lein M. (2016). Choosing the right precursor for thermal decomposition solution-phase synthesis of iron nanoparticles: tunable dissociation energies of ferrocene derivatives. *Physical Chemistry Chemical Physics*.

[18] Muzami M., Khalid N., Aziz M. D., Abbas S. A. (2014). Synthesis of silver nanoparticles by silver salt reduction and its characterization. *IOP Conference Series: Materials Science and Engineering*.

[19] Masala O., Seshadri R. (2004). Synthesis routes for large volumes of nanoparticles. *Annual Review of Materials Research*.

[20] Latha C. K., Raghasudha M., Aparna Y., Ravinder R. M. D., Veerasomaiah J. K. P., Shridhar D. (2017). Effect of capping agent on the morphology, size and optical properties of In_2_O_3_ nanoparticles. *Materials Research*.

[21] Salavati-Niasari M., Davai F., Mazaheri M. (2008). Synthesis of Mn_3_O_4_ nanoparticles by thermal decomposition of a [bis(salicylidiminato)manganese(II)] complex. *Polyhedron*.

[22] Betancourt-Galindo R., Retes-Rodriguez P. Y., Puente-Urbina B. A. (2014). Synthesis of copper nanoparticles by thermal decomposition. *Journal of Nanomaterials*.

[23] Wang Y., Xia Y. (2004). Bottom-up and top-down approaches to the synthesis of monodispersed effect of capping agent on the morphology, size and optical properties of In_2_O_3_ nanoparticles spherical colloids of low melting-point metals. *Nano Letters*.

[24] Behari J. (2010). Principles of nanoscience: an overview. *Indian journal of experimental biology*.

[25] Pomogalio D., Dzhardimlieva G. I. (2017). *Nanostructured Materials Preparation Via Condensation Ways*.

[26] Tran Q. H., Nguyen V. Q., Le A. (2013). Silver nanoparticles: synthesis, properties, toxicology, applications and perspectives. *Advances in Natural Sciences: Nanoscience and Nanotechnology*.

[27] Palacious-Hernández T., Hirata-Flores G. A., Contreras-López O. E. (2012). Synthesis of Cu and Co metal oxide nanoparticles from thermal decomposition of tartrate complexes. *Inorganica Chimica Acta*.

[28] Kino T., Kuzuya T., Itoh K., Sumiyama K., Wakamatsu T., Ichidate M. (2008). Synthesis of chalcopyrite nanoparticles via thermal decomposition of metal-thiolate. *Materials Transactions*.

[29] Rao C. N. R., Thomas P. J., Kulkami G. G. U. (2007). Nanocrystals: synthesis, properties and applications. *Springer Series in Materials Science*.

[30] Yeap S. P. (2018). Permanent agglomerates in powdered nanoparticles: formation and future prospects. *Powder Technology*.

[31] Rezende C. P., Da Silva J. B., Mohallem N. D. S. (2009). Influence of drying on the characteristics of zinc oxide nanoparticles. *Brazilian Journal of Physics*.

[32] Sabir S., Arshad M., Chaudhari S. K. (2014). Zinc oxide nanoparticles for revolutionizing agriculture: synthesis and applications. *Scientific World Journal*.

[33] Wostek-Wojciechowska D., Jeszka J. K., Uznanski P., Amiens C., Chaudret B., Lecante P. (2004). Synthesis of gold nanoparticles in solid state by thermal decomposition of an organometallic precursor. *Materials Science Poland*.

[34] Dallas P., Bourlinos A. B., Komninou P., Karakassides M., Niarchos D. (2009). Silver nanoparticles and graphic carbon through thermal decomposition of a silver/acetylenedicarboxylic salt. *Nanoscale Research Letters*.

[35] Jung Y. K., Kim J. I., Lee J. (2010). Thermal decomposition mechanism of single-molecule precursors forming metal sulfide nanoparticles. *Journal of the American Chemical Society*.

[36] Khalil M., Al-Qunaibitt M. M., Al-zahem A. M., Labis J. P. (2014). Synthesis and characterization of ZnO nanoparticles by thermal decomposition of a curcumin zinc complex. *Arabian Journal of Chemistry*.

[37] Nassar M. Y., Ahmed I. S., Samir I. (2014). A novel synthetic route for magnesium aluminate (MgAl_2_O_4_) nanoparticles using sol-gel auto combustion method and their photocatalytic properties. *Spectrochimica Acta Part A: Molecular and Biomolecular Spectroscopy*.

[38] Krishnaiah M., Bhargava P., Sudhanshu M. (2015). Low-temperature synthesis of Cu_2_CoSnS_4_ nanoparticles by thermal decomposition of metal precursors and the study of its structural, optical and electrical properties for photovoltaic applications. *RSC Advances*.

[39] Singh S., Bharti A., Meena V. K. (2014). Structural, thermal, zeta potential and electrical properties of disaccharide reduced silver nanoparticles. *Journal of Materials Science: Materials in Electronics*.

[40] Baldan A. (2002). Review progress in Ostwald ripening theories and their applications to nickel-base superalloys. Part 1: Ostwald’s ripening theories. *Journal of Materials Science*.

[41] Zhang Z., Wang Z., He S., Wang C., Jin M., Yin Y. (2015). Redox reaction induced Ostwald ripening for size-and shape-focusing of palladium nanocrystals. *Chemical Science*.

[42] Buzea C., Blandino I. I. P., Robbie K. (2007). Nanomaterials and nanoparticles: sources and toxicity. *Biointerphases*.

[43] Durán N., Marcato P. D., De Conti R. (2010). Potential use of silver nanoparticles on pathogenic bacteria, their toxicity and possible mechanisms of action. *Journal of the Brazilian Chemical Society*.

[44] Elsaesser A., Howard C. V. (2012). Toxicity of nanoparticles. *Advanced Drug Delivery Reviews*.

[45] Ravishankar R. V., Jamuna B. A. (2011). Nanoparticles and their potential application as antimicrobials. *Formatex*.

[46] Kalantzi O., Biskos G. (2014). Methods for assessing basic particle properties and cytotoxicity of engineered nanoparticles. *Toxics*.

[47] Love S. A., Maurer-Jones M. A., Thompson J. W., Lin Y. (2012). Assessing nanoparticles toxicity. *Annual Review of Analytical Chemistry*.

[48] Bahadar H., Maqbool F., Niaz K., Abdollahi M. (2016). Toxicity of nanoparticles and an overview of current experimental models. *Iranian Biomedical Journal*.

